# Machine Learning‐Based Multi‐omic Analyses of UK Biobank Identify Genes and Pathways Implicated in Alzheimer's Disease

**DOI:** 10.1002/alz70855_104450

**Published:** 2025-12-24

**Authors:** Maryam Samieinasab

**Affiliations:** ^1^ Baylor College of Medicine, Houston, TX, USA

## Abstract

**Background:**

Alzheimer's disease (AD) is a late‐onset neurodegenerative disorder with high genetic heritability (∼85%). However, genome‐wide association studies (GWAS) explain only a fraction of this heritability. Protein expression, influenced by cis‐ and trans‐regulatory effects, may contribute to the unexplained heritability and provide insights into AD biology.

**Method:**

We hypothesized that proteomic data from the UK Biobank (UKB) could capture protein expression differences between AD cases and healthy controls. Using OLINK proteomics data on 2,848 proteins from 537 AD cases and matched controls, we applied an ensemble machine learning (ML) approach, training nine ML classifiers to rank proteins based on their ability to distinguish AD cases from controls. To integrate genetic data, we incorporated whole exome sequencing (WES) variants, annotated with evolutionary action (EA) scores, to estimate the mutational effects of all variants within a gene for each individual. These genetic scores were combined with protein expression data as training features to identify AD candidate genes and assess their predictive value for AD diagnosis.

**Result:**

Our ML model identified 35 proteins, which can separate AD cases from controls based on expression alone, including APOE, NEFL, and GFAP. STRING protein‐protein interaction network analysis revealed that these 35 proteins formed significant clusters (*p* = 2×10⁻^8^) and were enriched in AD‐related pathways. Additionally, the 35 proteins significantly connected to established AD gold‐standard (GS) genes (*AUC* = 0.94, *Z* = 7), suggesting functional relevance. Additionally, WES analysis identified 153 candidate genes, which clustered significantly within the STRING network (*p* = 10⁻^4^). These genes replicated known AD GWAS hits (*p* = 3×10⁻^5^) and exhibited significant connectivity to AD GS genes (AUC = 0.78, Z = 3.1). These findings suggest that the identified proteins may influence, regulate, or interact with key genes implicated in AD biology.

**Conclusion:**

This study uncovers a potential proteomic architecture underlying AD. Further, integrating the genetic variants with the proteomics demonstrate the interplay between genetic variants and protein expression in the AD mechanisms and pathways. Future efforts will refine this framework to identify gene variants and proteomic signatures, advancing the discovery of therapeutic targets for AD.

